# Comparative efficacy of non-sedating antihistamine updosing in patients with chronic urticaria

**DOI:** 10.1186/1939-4551-7-33

**Published:** 2014-11-26

**Authors:** Mario Sánchez-Borges, Ignacio Ansotegui, Jorge Montero Jimenez, Maria Isabel Rojo, Carlos Serrano, Anahí Yañez

**Affiliations:** Allergy and Clinical Immunology Department, Centro Médico-Docente la, Trinidad and Clínica El Avila, 6a transversal Urb. Altamira, piso 8, consultorio 803, Caracas, 1060 Venezuela; Department of Allergy and Immunology, Hospital Quirón Bizkaia, Erandio, Spain; Unidad de Alergia, Hospital Mexico, CCSS, San Jose, Costa Rica; Allergy, Juarez Hospital, Mexico City, Mexico; Allergy Unit, Hospital Fundación Valle del Lili, Cali, Colombia; Investigaciones en Alergia y Enfermedades Respiratorias, InAER, Buenos Aires, Argentina

**Keywords:** Angioedema, Antihistamines, Chronic urticaria

## Introduction

Urticaria and angioedema lasting more than 6 weeks have been designated as chronic urticaria (CU). It encompasses two major subtypes: chronic spontaneous urticaria (CSU) (previously known as chronic idiopathic urticaria) (CIU) and chronic inducible urticaria. CSU has been defined as wheals and/or angioedema that are endogenous and independent of any external physical stimulus. It affects 0.5 to 1% of the population [1]. In 40 to 45% of patients with CSU autoantibodies to the high affinity IgE receptor (FcϵRI) or to IgE itself are thought to play a psathogenic role, whereas 55 to 60% of cases are considered idiopathic [2]. Inducible urticarias include all forms of physical urticarias (cold-induced, heat-induced, solar, and pressure urticaria).

According to the International Guidelines for the management of urticaria and angioedema non-sedating, second generation antihistamines (NSAHs) are recommended for the treatment of CU [3]. Nevertheless, a considerable proportion of patients do not respond sufficiently to NSAHs. According to Humphreys and Hunter up to 40% of patients with CU may not achieve good control with antihistaminic therapy [4]. They reported that out of 390 CU patients who were treated with antihistamines 44% responded well, 29% became asymptomatic, and 15% showed partial improvement. In a recent paper from Japan it was observed that the improvement rates (defined as a urticaria symptom score UAS ≤ 3) in 117 CU patients who received standard doses of AHs were 36.6% at 12 months, 51.2% at 24 months, and 66.1% at 60 months, while the remission rates were 11.5%, 13.9%, and 27.7%, respectively [5].

In patients that do not respond to standard doses, the next step in guideline-based therapy is to increase AH doses up to 4 times [3]. Investigations assessing the response to various NSAHs have demonstrated that up-dosing is significantly more effective in reducing symptoms of CU than standard-dose treatment [6]. According to Kaplan, high-dose antihistamines are effective in 45-60% of patients with CSU [7], while about one third are antihistamine resistant regardless of which dose is used [8, 9].

The present article is a review of the literature on the treatment of CU with increased doses of NSAHs in order to investigate if there are differences in efficacy between the various second generation AHs that have been studied in controlled protocols. It must be noticed, however, that it is difficult to find clinical investigations that strictly follow the criteria recommended by the guidelines on the management of urticaria, and therefore studies included in this review were those in which higher doses of NSAHs were used regardless of the clinical response to conventional doses.

AHs included in this review are desloratadine, levocetirizine, fexofenadine, and the recently introduced NSAHs rupatadine and bilastine. Bilastine belongs to the piperidine class of antihistamines as do loratadine, desloratadine, and fexofenadine. Like other antihistamines bilastine is an H1 receptor inverse agonist. *In vitro* studies have shown that bilastine has a high specific affinity for the H1-receptor but it has no or very low affinity for 30 other tested receptors. The affinity for the H1 receptor is 3 and 6 times higher than for cetirizine and fexofenadine, respectively [10, 11]. Rupatadine fumarate is a new potent, long acting, orally active dual antagonist of both histamine H1 and Platelet-Activating Factor (PAF) receptors. In *in vivo* and *in vitro* studies rupatadine was as potent or even more potent than other second generation antihistamines (loratadine, terfenadine and cetirizine) or selective PAF antagonists [12].

## Methods

A literature search of PubMed/MEDLINE looking specifically at the studies that investigated the effects of increased doses of NSAHs in patients with all subtypes of CU was conducted. For analysis of the efficacy, only double-blind, placebo-controlled studies were selected, whereas uncontrolled studies were excluded.

Data on study drug, doses, study design, treatment duration, subtype of urticaria being treated, number of patients, and main parameter of efficacy, were collected. When available, efficacy data were pooled from different studies that utilized the same drug dose. The proportions of patients responding to the therapy were compared using the Fisher’s exact test with a significance level of p < 0.05.

## Results

Twelve studies that investigated the effects of higher doses of NSAHs were identified in this search. Among those, 3 papers dealing with the treatment of patients with CSU were excluded from analysis because of their open design, 2 employing cetirizine and one that utilized ebastine [13–15]. Another study by Metz et alwas also excluded because it assessed exclusively the effects of a 20 mg dose of rupatadine in patients with acquired cold urticaria whereas no comparisons with other doses of the drug were done [16].

Table 1 summarizes the details from 8 double-blind, placebo-controlled studies included in this report. Two investigations used fexofenadine, rupatadine, or desloratadine, and one study was done with levocetirizine or bilastine. In most studies NSAHs were administered for 28 days, although in the papers by Siebenhaar (with desloratadine) and Krause (with bilastine) the drugs were given for 7 days. Six articles included patients with CSU/CIU and other 2 studied patients with acquired cold urticaria. Four investigations chose mean pruritus scores as the main outcome, and the other 4 utilized the percentage of symptom-free patients as the main parameter of efficacy.

**Table 1:** **Studies included in this comparative analysis**

Author	Year	Drug	Study design	Treatment duration (days)	Urticaria subtype	n	Parameter of efficacy	Reference numbers
Finn	1999	Fexofenadine	DB,PC	28	CSU/CIU	439	MPS	17
Nelson	2000	Fexofenadine	R,DB,PC	28	CSU/CIU	418	MPS	18
Giménez-Arnau	2007	Rupatadine	R,DB,PC	28	CSU/CIU	329	MPS	19
Dubertret	2007	Rupatadine	R,DB,PC	28	CSU/CIU	277	MPS	20
Siebenhaar	2009	Desloratadine	R,DB,PC	7	ACU	30	% SF	21
Staevska	2010	Desloratadine	DB,PC	28	CSU/CIU	40	% SF	22
Staevska	2010	Levocetirizine	DB,PC	28	CSU/CIU	40	% SF	22
Krause	2013	Bilastine	R,DB,PC	7	ACU	20	% SF	23

*DB* double-blind, *PC* placebo-controlled, *R* randomized.

*CSU* chronic spontaneous urticaria, *CIU* chronic idiopathic urticaria, *ACU* acquired cold urticaria.

*MPS* mean pruritus score, *% SF* percentage of symptom-free patients.

Table 2 presents the results of the 8 studies in regard to efficacy of the treatment. It can be observed that the proportion of symptom improvement was highly variable, ranging from 3.4% to 71.6%, depending on the drug and dose. The best responses were obtained with fexofenadine, rupatadine, and bilastine.

**Figure 1:**
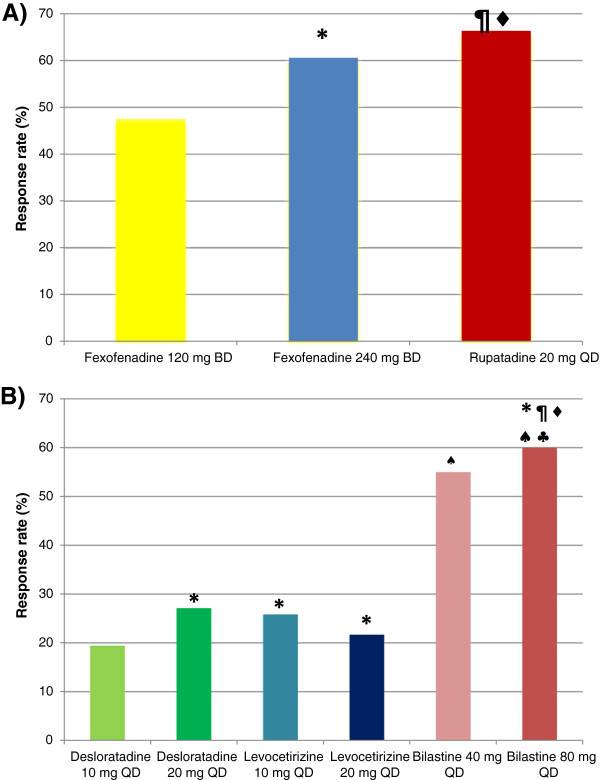
**Efficacy of increased doses of nonsedating antihistamines in patients with chronic urticaria. A)** According to mean pruritus score (MPS). *Fexofenadine 120 mg vs Fexofenadine 240 mg p = 0.01, ¶ Fexofenadine 120 mg vs Rupatadine 20 mg p = 0.0001, ♦ Fexofenadine 240 mg vs Rupatadine 20 mg p = 0.03. **B)** According to percentage of symptom-free patients. * Desloratadine 10 mg vs Desloratadine 120 mg, Desloratadine 10 mg vs Levocetirizine 10 mg, Desloratadine 10 mg vs Levocetirizine 20 mg, Desloratadine 20 mg vs Levocetirizine 10 mg, Desloratadine 20 mg vs Levocetirizine 20 mg, Levocetirizine 10 mg vs Levocetirizine 20 mg, Bilastine 40 mg vs Bilastine 80 mg p n.s. ¶ Desloratadine 10 mg vs Bilastine 40 mg p = 0.006. ♦ Desloratadine 10 mg vs Bilastine 80 mg p = 0.002. ♠ Desloratadine 20 mg vs Bilastine 40 mg, Desloratadine 20 mg vs Bilastine 80 mg, Levocetirizine 10 mg vs Bilastine 40 mg p = 0.02. ♣ Levocetirizine 10 mg vs Bilastine 80 mg p = 0.01.

The statistical comparison of the data is shown in Figure 1. There were not significant differences in efficacy between fexofenadine and bilastine, rupatadine and bilastine, and desloratadine and levocetirizine. However, fexofenadine, rupatadine, and bilastine showed significantly higher efficacy than desloratadine or levocetirizine, and rupatadine had higher efficacy than fexofenadine.

**Table 2:** **Efficacy of increased doses of non-sedating antihistamines in patients with chronic urticaria**

			Efficacy	
Author ^ref^	Drug	Dose (mg)	Responders/n	%
Finn [17]	Fexofenadine	120 BD	46/89	51.6
Finn [17]	Fexofenadine	240 BD	54/83	64.9
Nelson [18]	Fexofenadine	120 BD	33/77	42.8
Nelson [18]	Fexofenadine	240 BD	46/82	56.0
Giménez-Arnau [19]	Rupatadine	20 QD	69/109	63.3
Dubertret [20]	Rupatadine	20 QD	48/67	71.6
Siebenhaar [21]	Desloratadine	20 QD	15/30	50.0
Staevska [22]	Desloratadine	10 QD	7/36	19.4
Staevska [22]	Desloratadine	20 QD	1/29	3.4
Staevska [22]	Levocetirizine	10 QD	8/31	25.8
Staevska [22]	Levocetirizine	20 QD	5/23	21.7
Krause [23]	Bilastine	40 QD	11/20	55.0
Krause [23]	Bilastine	80 QD	12/20	60.0

## Discussion

According to current recommendations, patients with CU who do not respond to licensed doses of NSAHs should be switched to higher doses in order to obtain a better disease control. A number of publications that evaluated different NSAHs in increasing doses have clearly demonstrated that a higher proportion of patients previously uncontrolled exhibit significant improvements of their symptoms after going through this approach [6]. It is important to mention that these enhanced results have generally been accomplished without compromising patient’s safety, since no increased rates of side effects, including somnolence, have been observed.

The mechanisms explaining patient’s benefits from up-dosing are not completely understood, but increased *in vivo* receptor occupancy [24, 25], and effects of antihistamines on additional receptors have been proposed [26]. Observed differences in response to different NSAHs cannot be explained by terminal elimination half-life, duration of action, higher tissue/plasma concentration ratios or the presence of active metabolites in the skin [27]. An alternative hypothesis would be a differential H1-receptor occupancy by free (unbound) H1 antihistamine [25, 28]. The results discussed in present paper are in agreement with a previous report by Church and Maurer [29] who proposed that although the Ki may be an indicator of anti-H1 antihistamine potency *in vitro*, the large differences in volume of distribution and tissue accumulation in humans preclude this from being a good predictor of clinical efficacy in CSU.

In a previous review article we had proposed that favorable responses to high doses of NSAHs in patients with CU were not uniformly observed, and it was likely that there would be dissimilar results when outcomes from different studies were compared [6]. Present article shows that in fact some higher doses of NSAHs, notably fexofenadine, rupatadine, and bilastine, induced better objective improvements than desloratadine and levocetirizine (Table 2, Figure 1). The reasons for these differences are not clear at this time, but may depend on differential properties of the drugs, such as their chemical structure, *in vivo* anti-inflammatory actions, metabolism, blockade of various receptors, and interactions with transporter systems (e.g., P-glycoprotein) [30]. In the case of fexofenadine, however, two studies demonstrated that higher doses were not more efficacious than the standard 60 mg twice a day dose [17, 18].

Since more than 30% of CU patients are refractory to antihistamine therapy, additional pharmacological strategies are available. Alternative drugs inducing better responses in AH-resistant CU, as based on scientific evidences, include the addition of leukotriene receptor antagonists, corticosteroids, cyclosporine, or omalizumab [7, 31, 32]. The choice of alternative, off-label agents, should be based on availability, relative safety, and socioeconomic considerations.

When administering high doses of antihistamines questions on their safety are usually put forward. Studies conducted up to now have not demonstrated important concerns on predictable or newer adverse effects of up to 4 times recommended doses of NSAHs. Headache was the most frequent adverse effect reported for fexofenadine [17] and rupatadine [19], but its incidence was not higher than in placebo-treated patients. Somnolence, drowsiness, or sedation was uncommon, although for patients treated with rupatadine 20 mg somnolence was observed more often than in the placebo group in two studies [19, 20]. The utilization of increased doses of desloratadine, levocetirizine, and bilastine has not been associated with adverse effects. Additionally, Staevska et al reported that patients taking higher doses of levocetirizine or desloratadine showed a paradoxical decrease in somnolence that was attributed to the relief from urticaria-related discomfort leading to a better quality of sleep although an alternative explanation would be the development of tolerance to the central nervous sedative effects of the antihistamines [22].

The results presented in this paper must be taken into consideration cautiously because there is a large heterogeneity between studies included in regard to various aspects of the investigation such as the subtype of chronic urticaria under study, duration of the treatment, study design, drug doses, and primary outcomes.

We can conclude that increased doses of NSAHs show an improved efficacy in patients with CU who do not respond to approved doses. According to the studies presented in this paper, this conclusion would be applicable to CSU/CIU and acquired cold urticaria, but more research would be necessary to be able to elucidate if this approach is valid for other types of urticaria. There are differences in efficacy of these drugs that should be taken into account in the clinical setting. The use of double approved doses of fexofenadine, rupatadine, or bilastine shows an objective improvement in most (>50%) of patients that respond to antihistamines. Desloratadine requires four times the approved dose to reach similar results.

There is still the need for additional studies designed to investigate the response to high doses of NSAHs in patients who do not respond to recommended doses, adapted to current guideline recommendations.
